# Accessible Dyslexia Detection with Real-Time Reading Feedback through Robust Interpretable Eye-Tracking Features

**DOI:** 10.3390/brainsci13030405

**Published:** 2023-02-26

**Authors:** Ivan Vajs, Tamara Papić, Vanja Ković, Andrej M. Savić, Milica M. Janković

**Affiliations:** 1 School of Electrical Engineering, University of Belgrade, Bulevar Kralja Aleksandra 73, 11120 Belgrade, Serbia; 2 Innovation Center, School of Electrical Engineering, Bulevar Kralja Aleksandra 73, 11120 Belgrade, Serbia; 3 Faculty of Technical Sciences, University Singidunum, Danijelova 32, 11000 Belgrade, Serbia; 4 Faculty of Philosophy, University of Belgrade, Čika-Ljubina 18-20, 11000 Belgrade, Serbia

**Keywords:** dyslexia, reading, eye tracking, feature extraction, machine learning, statistical analysis, colors

## Abstract

Developing reliable, quantifiable, and accessible metrics for dyslexia diagnosis and tracking represents an important goal, considering the widespread nature of dyslexia and its negative impact on education and quality of life. In this study, we observe eye-tracking data from 15 dyslexic and 15 neurotypical Serbian school-age children who read text segments presented on different color configurations. Two new eye-tracking features were introduced that quantify the amount of spatial complexity of the subject’s gaze through time and inherently provide information regarding the locations in the text in which the subject struggled the most. The features were extracted from the raw eye-tracking data (x, y coordinates), from the original data gathered at 60 Hz, and from the downsampled data at 30 Hz, examining the compatibility of features with low-cost or custom-made eye-trackers. The features were used as inputs to machine learning algorithms, and the best-obtained accuracy was 88.9% for 60 Hz and 87.8% for 30 Hz. The features were also used to analyze the influence of background/overlay color on the quality of reading, and it was shown that the introduced features separate the dyslexic and control groups regardless of the background/overlay color. The colors can, however, influence each subject differently, which implies that an individualistic approach would be necessary to obtain the best therapeutic results. The performed study shows promise in dyslexia detection and evaluation, as the proposed features can be implemented in real time as feedback during reading and show effectiveness at detecting dyslexia with data obtained using a lower sampling rate.

## 1. Introduction

The definition of dyslexia has been a topic in many research studies [1,2,3], but regardless of the definition, its detrimental effects on academic performance [4,5] and quality of life [6,7,8] have been consistently proven. Multiple studies have the goal of improving the understanding of dyslexia from multiple different aspects, including neural activity [9,10], visual sampling strategy [11,12,13], emotional response [14,15], educational performance [16,17], social status [18,19], and others [20,21,22,23]. However, considering the nature of experimental and study requirements, implementations of the study results for assistive and treatment purposes often prove to be a challenge in itself [24,25,26].

Although different aspects of dyslexia are studied in the literature, in practice, the diagnosis of dyslexia is commonly based on standardized oral or written tests conducted by specialists. These tests take into consideration background information, intelligence, oral and reading language skills, as well as other relevant tests [27]. With the advancement of technologies, however, different diagnostical procedures are being proposed in the literature, focusing on different measurable and quantifiable criteria based on biomarker approaches [28,29].

The visual sampling strategy of dyslexia (i.e., gaze patterns) is often analyzed in dyslexia research for the purposes of dyslexia diagnosis and evaluation. The irregularity in gaze patterns of dyslexic subjects has long been analyzed in studies [30,31], but technological advancement has made dyslexia eye-tracking studies more common and the experimental setup scenarios more diverse. In Rello et al. [32], the authors have reportedly performed a first study focusing on dyslexia classification based on the eye event (fixations and saccades) parameters during reading. A Tobii 1750 eye tracker was used to measure the eye movements of 98 subjects (48 dyslexic), and a support vector machine (SVM) was used to differentiate dyslexic and control subjects based on derived features and participant characteristics. This study included native Spanish speakers and reported an accuracy of 80.18% and has paved the way for many future similar studies. In [33], the authors monitor the eye movements of 185 subjects (97 dyslexic) using a goggle-based Ober-2 eye-tracker. The study included participants with ages ranging from 9 to 10, presented them a text on a piece of paper, and derived 168 eye-tracking features from the recorded data. They implemented recursive feature elimination (RFE) to reduce the number of features and trained an SVM classifier to detect dyslexic reading patterns. The results were an accuracy of 95.6%, showing the methodology as quite effective. Prabha et al. in [34] also analyzed the dataset obtained in [33], focusing on only the features extracted from fixations. The authors implanted an RFE feature selection algorithm and several different machine learning (ML) algorithms, including SVM, k-nearest neighbors (KNN), and random forest (RF). They obtained an accuracy of 95% and continued their work through several other publications, analyzing the same dataset [35,36,37]. They tried different ML algorithms and eye-tracking feature inputs, obtaining a slightly higher accuracy of 96% in [36], and providing a method for clustering the dyslexic group into high and low dyslexics, providing reference ranges for fixation and saccade duration to estimate the severity of dyslexia [37].

In [38], a study including 69 children (32 dyslexic), ages 8.5–12.5, was performed. The study included native Greek speakers, and the participants were given two paragraphs of text to read while their eye movements were recorded with a custom eye tracker. The study employed several ML algorithms and a multitude of features, obtaining an accuracy of 97% with only three features: saccade length, the number of short forward movements, and the number of repeatedly fixated words. In [39], the authors used an EyeLink 1000 eye tracker to monitor the eye movements of 165 (30 dyslexic) Finnish students with an average age of 12.5. The authors implemented two ML algorithms, SVM and RF, and obtained an accuracy of 89.7% using a novel, transition-matrix-based feature extraction. Observing the students during both reading and non-reading tasks, El Hmimdi et al. analyzed 87 subjects (46 dyslexic) recruited from schools in Paris [40]. Two non-reading and one reading test were performed, and the eye movements were monitored using a Pupil Core head-mounted video-oculography device. Based on a variety of eye-tracking features and ML algorithms, the authors report an accuracy of 81.25% from the reading tests and 81.25% and 77.3% from the two non-reading tests. A study including 30 Serbian children (15 dyslexic) was performed in [41], focusing on the spatio-temporal characteristics of gaze during reading. The experiment included the reading of 13 text segments, each presented on a different background/overlay color, and used a SMI RED-m portable remote eye tracker to monitor the participants’ eye movements. The study implemented several ML algorithms using various features individually as well as feature groups as inputs and obtained an accuracy of 94% for a combination of features, with an accuracy of 91% for a single feature describing the spatial complexity of gaze.

Recent studies also report attempts at detecting dyslexia from eye tracking during reading without performing feature extraction rather than working with minimally processed data using convolutional neural networks (CNNs). In [42], the authors used the dataset from [33] and implemented a one-dimensional neural network that performed dyslexia detection on the sequences of x and y gaze coordinates. The study reports an accuracy of 96.6%, showing that a more holistic approach can indeed be used to detect dyslexic tendencies. The authors in [43] also perform dyslexia classification using a CNN, but use 2D images as inputs instead. The study was performed using data gathered in [44] and used color coding to represent gaze data in the form of colored images. These images were then used to train and evaluate a deep two-dimensional CNN, obtaining an accuracy of 87% and proving that the spatial characteristics of gaze carry enough information for deep learning algorithms to provide accurate dyslexia detection. A method that also used CNN and the spatial characteristics of gaze for dyslexia detection was presented in [45]. The method does include preprocessing steps and feature extraction, but it does not use eye-event parsing, and it was implemented and tested on two datasets (trained on one and tested on the other) from [44] and [33], achieving accuracies of 85.6% and 82.9%, respectively.

The abundance of eye-tracking studies in dyslexia points to the fact that a dyslexia diagnosis can indeed be successfully made based on eye tracking during reading. However, implementation of the scientific results in terms of eye tracking during reading, in practice, is not abundantly present. There are tools reported in the scientific literature that focus on the digitalization of dyslexia diagnosis [46,47], some of which do use eye tracking [38], but considering the variety of study conditions including eye trackers used, text presentation, native language, participant age, and many others, this makes practical applications somewhat complicated and difficult. Providing feedback during reading in terms of exact points in the text that caused struggles would also be quite difficult, considering that few eye-tracking processing techniques can pinpoint the locations in the text that were problematic to read.

Finally, examining the impact of colors on reading performance is challenging in research as well as in practice [48]. The use of colored overlays, lenses, plastic sheets, and visual changes in the presentation of text, such as background color and font size, can relieve dyslexia symptoms [49,50]. Some studies have suggested that certain colors (like peach, orange, yellow, turquoise, and so on) may be more effective than others in reducing visual stress and improving reading performance in dyslexics [44,51]. Therefore, the effect of colors should certainly be taken into consideration when designing studies dealing with the analysis of reading difficulties.

The goal of the study was the creation of eye-tracking features that could differentiate between dyslexic and control subjects, work on low-frequency eye trackers, and be independent of eye movement parsing (fixations and saccades). The proposed features inherently provide locations in the text where the struggle during reading was detected, making real-time feedback possible. Furthermore, the study performs an analysis of the influence of color configuration (CC) on the performance of subjects, showing the importance of the presentation of the text and its possible impact on the dyslexia diagnosis and monitoring success.

## 2. Materials and Methods

### 2.1. Dataset

The data were acquired in a previous study [44] and included an experiment where Serbian children were instructed to read 13 text segments, each presented on a computer screen with different CC. The data analyzed in this study were gathered from 30 subjects (15 dyslexic), with ages ranging from 7 to 13, and the distribution of age is the same for both the dyslexic and control groups. Each text segment (2–3 sentences) is presented on a different CC, with the first always being black text on a white background and the others having a pseudo-random order, including the colors red, blue, yellow, orange, turquoise, and purple as both backgrounds for black text, and a transparent overlay over black text on a white background. The analyzed data included 378 trials, with a trial representing the reading of one text segment from one participant. The 378 trials were obtained from a total of 30 subjects×13 CC=390 trials, after excluding 12 trials owing to a short reading time (reading time less than 5 s), exhibiting insufficient focus on the text. The subjects were monitored during reading using a multimodal sensor hub [20], but only the eye-tracking data were used in this study. An SMI RED-m portable remote eye-tracker (iMotions, Copenhagen, Denmark) was used to record the eye movements and the data were acquired at a sampling frequency of 60 Hz.

### 2.2. Feature Extraction

The feature extraction process is designed to rely only on the sequence of x and y coordinates of gaze, without the information on eye event occurrence, i.e., fixations and saccade events. Firstly, the data points that had no information on the gaze coordinates for both eyes were removed, leaving only the data where the gaze could be located on the screen (the average percentage of data per trial that contained no gaze coordinates was 4.5% of total trial data for the dyslexic group and 2.5% for the neurotypical group). Secondly, two different methods for feature extraction, with the same goal but different characteristics, were implemented to detect the subjects’ struggle during reading (feature 1 and feature 2). The feature extraction was performed only on valid gaze data.

Feature 1 was based on the detection of the self-intersection (SI) of gaze lines during reading. Every four consecutive data points in the x–y coordinate plane are observed, and it was determined whether the gaze line connecting these points has an SI. If there is an intersection, the number of changes in the direction of gaze movement on the *y*-axis is monitored for the following 250 ms (approximately an average fixation duration [52]). Each change is counted, and a vertical alteration score (VAS)—indicating the number of changes in the direction of movement on the *y*-axis—is added to the total VAS of feature 1. So, feature 1 starts off at the value of 0 at the beginning of the trial, and each time an SI is detected in four consecutive data points, the feature can increase based on the VAS in the time period that follows the SI event. This effectively causes the feature value to progressively rise during the trial, but uses the SI event as a form of a trigger, and the VAS of the included time intervals as the quantification of struggle during reading.

Feature 2 follows the same principle as feature 1, but instead of using an SI of four consecutive data points as a trigger to add the VAS, it uses the number of changes in the gaze direction on the *y*-axis of four consecutive data points (observing the VAS in four consecutive data points). If there are two changes in the direction of movement on the *y*-axis in the four observed consecutive data points, the VAS of the gaze obtained from the 250 ms following the trigger event is added to the total VAS of feature 2.

**Figure 1:**
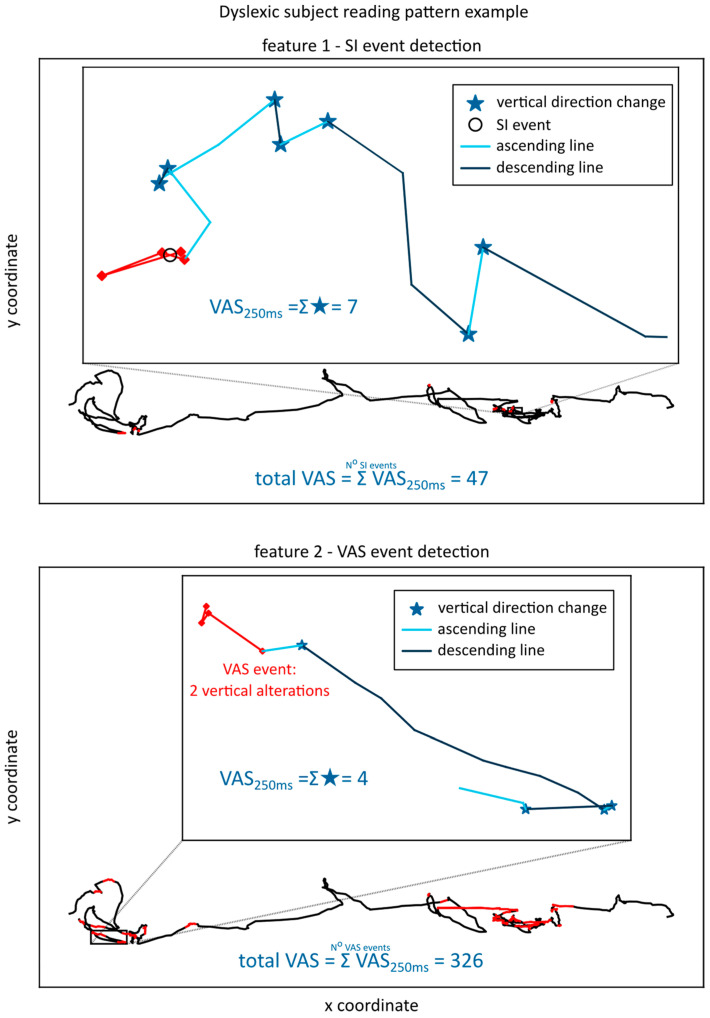
Feature 1—SI event detection and Feature 2—VAS event detection for a dyslexic subject, with the gaze lines corresponding to the trigger events colored red.

An example of feature 1 and feature 2 extracted for the reading of one line of text for a dyslexic subject is given in Figure 1.

**Figure 2:**
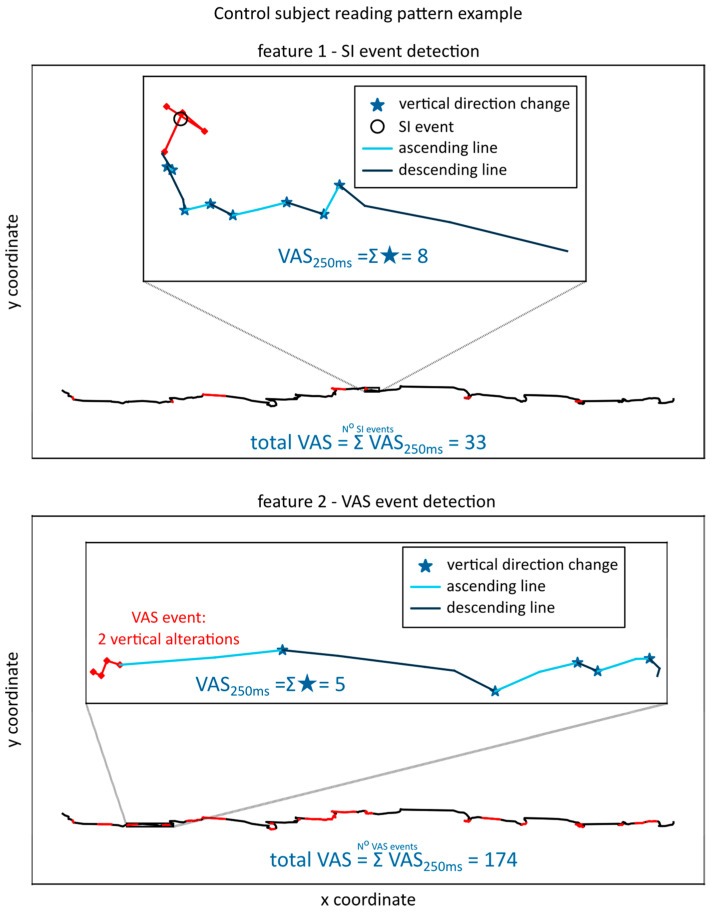
Feature 1—SI event detection and Feature 2—VAS event detection for a control subject, with the gaze lines corresponding to the trigger events colored red.

Figure 1 clearly shows a much higher number of trigger events in feature 2, as the change in the y direction is much more common than SI in four consecutive points. Consequentially, feature 2 will have much higher values than feature 1, detecting more events and thus having more opportunity to increase based on the VAS in the observed time interval of 250 ms. An example of the extraction of feature 1 and feature 2 for one line of text for a control subject is given in Figure 2.

In contrast to the dyslexic subject, the control subject has a lower number of trigger events and overall lower feature values. The VAS is not close to 0, especially when the trigger event is based on the VAS detection in four consecutive data points, because vertical changes in gaze movement are not indicative of a dyslexic reading pattern per se, but the number of changes after trigger events is expected to be lower in controls because dyslexic subjects do have a more spatially complex gaze pattern [41,45].

To investigate the stability of the proposed features and whether they could be used with data acquired at a lower sampling rate, the feature extraction process was repeated on gaze data downsampled from 60 Hz to 30 Hz. This would result in four total features observed in the paper: feature 1 on 60 Hz, feature 1 on 30 Hz, feature 2 on 60 Hz, and feature 2 on 30 Hz. Each of these was analyzed independently as inputs to various machine learning algorithms and through statistical analysis.

Additionally, the total reading time of each trial was used as input to the ML algorithms as a baseline feature for dyslexia classification.

### 2.3. Machine Learning and Statistical Analysis

Several different ML algorithms were implemented in this study, and each of them had only a single feature at a single sampling frequency as an input. Leave-one-subject-out evaluation was implemented to obtain results that would be similar those in practice, taking all the data from one subject and placing it in the test set and using the rest of the data as training. This was repeated 30 times (each subject was placed in the test set), and the predictions and labels of all the test sets were concatenated and used to provide classification metrics. The algorithms used were logistic regression (LR), SVM, KNN, and RF, and the metrics used were accuracy (ACC), sensitivity (Se), specificity (Sp), F1-score, and the area under the receiver operating curve (AUROC). For each training fold, a grid search for hyperparameter optimization was performed on the training set, using fivefold cross-validation.

The statistical analysis of the data includes a comparison between the dyslexic and control group on all collective CC, and for each CC separately, using the Mann–Whitney statistical test. An analysis of the impact of each CC on the dyslexic group was performed by comparing each pair of the CC within the dyslexic group, also using the Mann–Whitney test. The effect of the CC on the separability of classes was also analyzed by displaying the boxplots for each feature for the dyslexic and control groups when each of the individuals was evaluated on their best-performing CC and on their worst-performing CC.

The processing, ML algorithm implementation, statistical analysis, and visualization of the data were performed in the Python programming language, using the sklearn and matplotlib libraries [53,54,55].

## 3. Results

### 3.1. Classification Results

The results of the performed cross-validation for each feature and for all observed ML algorithms are shown in Table 1.

The evaluation results show several important findings. Firstly, regardless of the ML algorithm and the proposed features used as the input, the classification accuracy is above 82%, indicating that the features are overall effective at separating the dyslexic and control groups. The results also show an improved performance when compared with the considered baseline (total reading time). Secondly, the best results, in terms of ACC, are obtained for the VAS event detection feature at 60 Hz and for the LR and SVM, being 88.9%. Finally, the accuracies obtained for the features extracted at 30 Hz are not much lower than the values obtained for features extracted at 60 Hz, with the best result being an ACC of 87.8% for the VAS event detection feature and the LR algorithm. This is also equal to the best-obtained accuracy for the SI event detection feature, giving a slight advantage to the VAS detection in the classification performance.

### 3.2. Color Configuration—Reading Performance Results

The results of the statistical analysis are congruent with the overall classification results. There is a statistically significant difference (*p* < 0.001) between the dyslexic and control group when all CCs are observed together for each of the two features at both frequencies. When comparing the dyslexic and control group on each CC separately, there is a statistically significant difference (*p* < 0.001) for all CCs.

**Figure 3:**
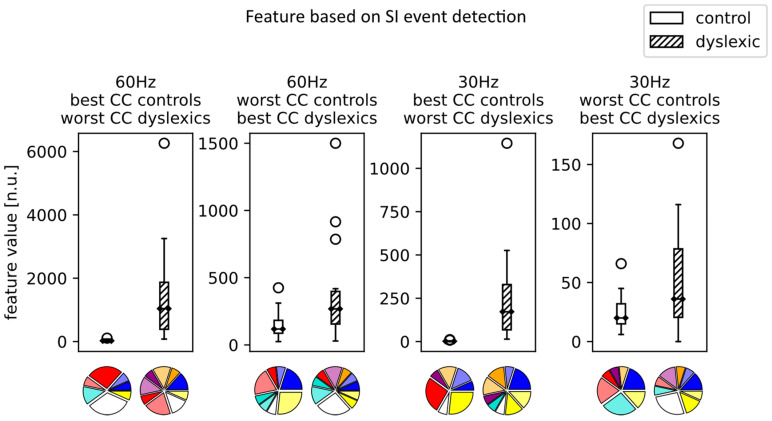
The boxplots showing SI event feature values for the maximally separated classes (control subjects evaluated on their best CC and the dyslexic subject evaluated on their worst CC) and the classes brought together (dyslexic subjects evaluated on their best CC and the control subject evaluated on their worst CC). Below each boxplot is a pie chart displaying the distribution of CCs that were used for the subjects’ feature values (best- or worst-performing CCs).

**Figure 4:**
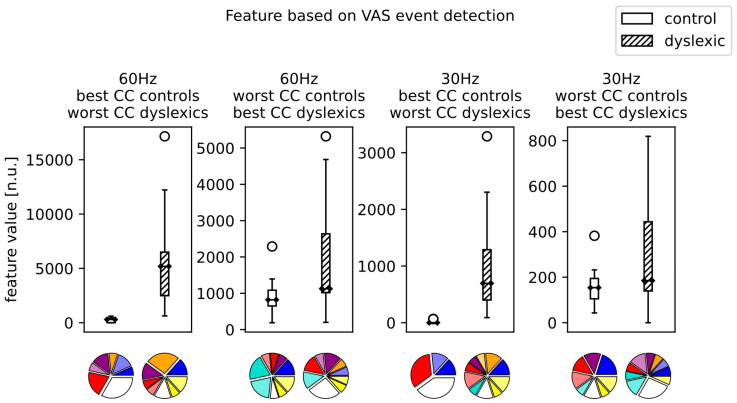
The boxplots showing VAS event feature values for the maximally separated classes (control subjects evaluated on their best CC and the dyslexic subject evaluated on their worst CC) and the classes brought together (dyslexic subjects evaluated on their best CC and the control subject evaluated on their worst CC). Below each boxplot is a pie chart displaying the distribution of CCs that were used for the subjects’ feature values (best- or worst-performing CCs).

Comparing the CCs within the dyslexic group suggested no statistically significant difference. This, however, does not mean that the influence of color is negligible, just that there is no single CC that is universally better than the others. The influence of CC can be seen in Figure 3 for the SI event detection and in Figure 4 for VAS event detection features, at both 30 and 60 Hz, when the dyslexic and control groups are evaluated on each individual subject’s best-/worst-performing CC.

### 3.3. Computational Resources

The feature extraction was executed on an Intel(R) Core(TM) i3-7100 CPU @ 3.90 GHz processor with 16 GB of RAM. The quotient of the total time needed for feature extraction and the total reading time of all subjects is 7.21‰ for the SI event feature and 7.33‰ for the VAS event feature at 60 Hz. Effectively, this means that, for 1 s of reading time, ≈7 ms of processing time is needed, making real-time processing and feedback possible. The processing time is halved for feature extractions performed for data downsampled to 30 Hz.

## 4. Discussion

When observing the obtained classification ACC, it is not above 90%, which is reported in several studies, e.g., [38,42], but it definitely falls in the range of ACCs (80% to 96.6% [32,33,38,40,41,42]) that are common for dyslexia detection based on eye tracking during reading. The results of the authors’ previous study [41] showed an ACC of 94% obtained when using a combination of features. However, when observing a single feature, the best ACC was 91% (which is 2% higher than the results obtained in this study). Using a single feature is an important goal in the context of accessible dyslexia detection and monitoring, because providing simple feedback and a quantitative measure of dyslexic tendencies is easier on a single-feature scale. Furthermore, the features proposed in [41] introduce a concept of the spatial characteristics of the gaze, but rely on the parsing of the gaze into fixations and saccades, which is common in the literature. One more important aspect of the proposed features is that they have trigger events and continual scoring, making real-time feedback possible, and the locations at which the feature gained most of its value (struggles in reading) are traceable.

The features proposed in this paper rely on spatial characteristics, which are shown to be indicative of dyslexic behavior in different languages [45]. Feature 1, relying on the detection of self-intersections in gaze for the trigger events, was introduced as a measure that would be quite restrictive to trigger events, because it does require a certain spatial resolution of the eye-tracking device in order to detect the events. On the other hand, feature 2 was introduced, focusing only on the detection of the changes of movement direction in on the *y* (vertical) axis. This feature is a lot less restrictive in comparison with the first one, and the changes in the direction of movement can often occur in both dyslexic and control subjects. Having this in mind, a large number of these alterations could be considered a more complex spatial pattern, and the goal was to analyze how these two features would compare in dyslexia detection.

Both features proposed in this paper were shown to be quite effective in separating the dyslexic and control group, and have been shown to be effective (best ACC of 87.8%) when implemented at a sampling frequency of 30 Hz. There is no clear prediction in terms of the impact a lower sampling rate can have on classification in the reported studies, but it is important to note that the sampling frequencies of the reported studies are all above 30 Hz: 50 Hz in [32], 60 Hz in [38], 100 Hz in [33], 200 Hz in [40], and 1000 Hz in [39]. Considering the advancements in computer vision and the possibilities for eye tracking based on web cameras [56,57,58], the results of this paper show promise in accessible dyslexia detection.

The features are designed in such a way that each detected trigger event has a quantifiable contribution to the total VAS score. A higher total VAS score indicates a reader with more difficulties, so, consequently, the trigger events (which can be located in the text) with the higher contribution to the total VAS score can be considered as points of struggle or at least the points in the text that have made the quantification of dyslexic tendency higher. The proposed features show the potential for identifying text locations where reading difficulties are visually manifested with or without compensatory strategies involved. More experimental studies are needed to differentiate between problems in reading and various compensatory strategies [59], which goes beyond the scope of this paper.

Finally, when considering accessible dyslexia detection, the design of screening can be relevant. The results of Figure 3 show the influence of CC selection on the separability of the dyslexic and control group for the SI event feature. The groups have barely any intersections in feature values when they are maximally separated (control subjects evaluated on their best CC and dyslexic subject evaluated on their worst CC), while they are much closer in values when they are brought together (dyslexic subjects evaluated on their best CC and control subject evaluated on their worst CC). These scenarios, of course, represent extreme cases. However, they emphasize the influence of the way text is presented. The pie charts also show a variety of CCs, indicating that no color can be singled out regarding reading facilitation and that the distribution of CCs can vary depending on the sampling frequency.

This is further backed up by the influence of CC shown for the VAS event detection features; Figure 4. One thing that stands out is that, although the VAS event detection feature and the SI event detection feature are conceptually quite similar, they show some differences in the best/worst CC for the dyslexic and control groups. For example, the white background is quite prominent for both features as the best CC for control subjects at 60 Hz. On the other hand, at 30 Hz, white is present as the best CC for controls for the VAS event feature, but is barely present in the distribution for the best CCs of controls for the SI event feature. This would emphasize the importance of repeated evaluations of each CC, as well as using multiple features, as they might represent different reading patterns. Although both features separate the dyslexic and control groups effectively, they might not be completely congruent when it comes to the best way of presenting text.

Figure 3 and Figure 4 visually demonstrate that the color configuration has an impact on the feature values. The appropriate selection of the color configuration could increase or decrease feature values; that is, depending on the choice of colors, dyslexics can be closer or further away from neurotypicals (Figure 3 and Figure 4 present only extreme cases of the color configuration selection). This conclusion about the individual’s affective reaction to colors could be useful not so much for the classification procedure itself, but for the planning of the treatment for dyslexics using color text modifications. Additionally, pie charts in Figure 3 and Figure 4 demonstrate the importance of a personalized approach in the color configuration selection.

The results of this study show that there is no universally facilitating or aggravating CC for presenting text, neither for the control group nor the dyslexic group. However, when given the opportunity to read on their best-/worst-performing CCs, the separability between classes can be influenced. The conclusions of this study stand, therefore, with those from the literature [41,60] in terms of the importance of the text presentation method and emphasize the importance of the evaluation of subjects using various scenarios in order to obtain an accurate diagnosis.

## 5. Conclusions

In this paper, two novel eye-tracking features were introduced that were used to differentiate between the dyslexic and control groups. The features were designed with interpretability and robustness in mind and were implemented only based on the x and y coordinates of gaze and extracted from the original data (60 Hz) as well as from downsampled data (30 Hz). The features were used as inputs to various ML algorithms for dyslexia classification, and an accuracy of 88.9% for 60 Hz and 87.8% for 30 Hz was obtained. Statistical analysis was performed as well, showing that the features were effective at separating the groups on each CC, but that no CC is universally superior to others. The features’ interpretability, computational efficiency, and robustness regarding sampling frequency give them great potential for accessible dyslexia screening, especially as they focus on the spatial characteristics of gaze, which have been shown to possibly be indicative of dyslexic reading patterns in different languages. Future work would include the implementation of features on a larger dataset, possibly a different experiment scenario involving the reading of a longer text including visual crowding effects, and an analysis of eye movements of different languages, as well as use of the developed features with custom-made eye trackers based on web cameras.

## Figures and Tables

**Table 1:** Evaluation metrics on the test set for each feature and for all observed ML algorithms.

Feature/Frequency	ML Algorithm			
	LR	SVM	KNN	RF
SI event detection/60 Hz				
ACC	0.870	0.878	0.865	0.868
Se	0.822	0.822	0.822	0.822
Sp	0.917	0.933	0.907	0.912
F1 score	0.861	0.869	0.856	0.859
AUROC	0.869	0.877	0.864	0.867
SI event detection/30 Hz				
ACC	0.860	0.860	0.831	0.823
Se	0.811	0.805	0.795	0.795
Sp	0.907	0.912	0.965	0.850
F1 score	0.850	0.849	0.821	0.814
AUROC	0.859	0.859	0.830	0.822
VAS event detection/60 Hz				
ACC	0.889	**0.889**	0.881	0.881
Se	0.849	**0.854**	0.859	0.859
Sp	0.927	**0.922**	0.902	0.902
F1 score	0.882	**0.883**	0.876	0.876
AUROC	0.888	**0.888**	0.881	0.881
VAS event detection/30 Hz				
ACC	**0.878**	0.860	0.862	0.860
Se	**0.859**	0.816	0.827	0.827
Sp	**0.896**	0.902	0.896	0.891
F1 score	**0.874**	0.851	0.855	0.852
AUROC	**0.878**	0.859	0.862	0.859
Total reading time				
ACC	0.769	0.796	0.746	0.738
Se	0.654	0.605	0.622	0.589
Sp	0.860	0.974	0.865	0.834
F1 score	0.727	0.742	0.706	0.674
AUROC	0.790	0.675	0.758	0.733

Values in bold represent the best obtained results for the 60 Hz and 30 Hz.

## Data Availability

No new data were created in this study.
